# Direct Optofluidic Measurement of the Lipid Permeability of Fluoroquinolones

**DOI:** 10.1038/srep32824

**Published:** 2016-09-08

**Authors:** Jehangir Cama, Michael Schaich, Kareem Al Nahas, Silvia Hernández-Ainsa, Stefano Pagliara, Ulrich F. Keyser

**Affiliations:** 1 Biological and Soft Systems, Cavendish Laboratory, University of Cambridge, JJ Thomson Avenue, Cambridge CB3 0HE, United Kingdom; 2 Department of Mechanical and Process Engineering, ETH Zurich, CLA H 1.1 Tannenstrasse 3, 8092 Zurich, Switzerland; 3 Jacobs University Bremen, Campus Ring 1, D-28759, Bremen, Germany; 4 Department of Biosciences, College of Life and Environmental Sciences, University of Exeter, Exeter EX4 4QD, United Kingdom

## Abstract

Quantifying drug permeability across lipid membranes is crucial for drug development. In addition, reduced membrane permeability is a leading cause of antibiotic resistance in bacteria, and hence there is a need for new technologies that can quantify antibiotic transport across biological membranes. We recently developed an optofluidic assay that directly determines the permeability coefficient of autofluorescent drug molecules across lipid membranes. Using ultraviolet fluorescence microscopy, we directly track drug accumulation in giant lipid vesicles as they traverse a microfluidic device while exposed to the drug. Importantly, our measurement does not require the knowledge of the octanol partition coefficient of the drug – we directly determine the permeability coefficient for the specific drug-lipid system. In this work, we report measurements on a range of fluoroquinolone antibiotics and find that their pH dependent lipid permeability can span over two orders of magnitude. We describe various technical improvements for our assay, and provide a new graphical user interface for data analysis to make the technology easier to use for the wider community.

A reduction in cell permeability to antibiotics is known to be an important biochemical mechanism of drug resistance in bacteria^1^,^2^. This problem is particularly acute in the case of Gram-negative pathogens, whose double membrane cell envelope provides a formidable barrier to the cellular entry of hydrophilic drugs^2^. Therefore, the study of antibiotic diffusion across bacterial membranes is of fundamental importance, both for the development of new drugs as well as for improving the efficacy of existing antibiotics.

It is also critical to study the role of environmental factors on drug permeability. For example, the pH of the surrounding environment plays a major role on a drug’s ability to permeate a lipid membrane – at different pH values, the drug molecule can exist in different charge states, which changes its lipophilicity^3^,^4^. This is of particular importance while studying the passage of a drug capsule through the human digestive tract – the pH changes from highly acidic in the stomach (1.0–2.5) to more neutral values (6.5–7.5) in the intestine^5^. The microenvironment around an infection site can thus greatly influence the effectiveness of a drug.

However, obtaining quantitative information about drug permeability into bacterial cells is difficult due to their small size, and also because changes in experimental conditions and techniques can lead to contrasting results^6^. Understanding the exact pathways of drug transport across the cell membrane is also difficult with living cells due to the lack of control over the system. However, it is known that the majority of drug transport occurs due to passive diffusion across the cell membrane^7^,^8^. We therefore use giant unilamellar vesicles (GUVs) as highly controlled lipid systems. GUVs are created by electro-formation and we thus have excellent control over their composition, facilitating the study of passive drug permeation in a highly specific manner.

Microfluidics offers a convenient platform by which drug transport studies can be performed in a high throughput manner. There are numerous microfluidic techniques to study drug transport across Caco-2 cells, which provide information about the intestinal absorption characteristics of a drug^9^,^10^,^11^. Other approaches have targeted the role of the endothelial barrier lining blood vessels in drug uptake, where microfluidics enables the study of drug permeability under effects such as fluid-flow induced shear stress^12^. In addition, lipid vesicles have been used in microfluidic devices to study membrane transport, both through the lipid membrane and through protein nanopores inserted into the lipid membrane^13^,^14^,^15^. These methods typically involve immobilizing the vesicles on chip and flowing in the drug; drug uptake into the vesicles is visualised using fluorescent reporters.

We previously developed an optofluidic assay where GUVs are mixed with an antibiotic using a T junction microfluidic chip, the chip being illuminated with ultraviolet (UV) light^4^. Under UV illumination, many antibiotic molecules are autofluorescent, and can thus be tracked in a label-free manner. By studying the increase in autofluorescence within the vesicles over time as they pass through the channel in the presence of the antibiotic, we can determine the permeability coefficient of the drug^4^. Importantly, the technique determines drug permeability directly without requiring the measurement of the octanol partition coefficient. Until now, we have used this technique to study the pH dependence of the transport of norfloxacin, a fluoroquinolone antibiotic, across lipid membranes^4^. Fluoroquinolones are broad spectrum antibiotics that target intracellular processes catalysed by DNA topoisomerases. Fluoroquinolones thus disrupt the DNA supercoiling process and inhibit bacterial cell division^16^. We have also studied the dependence of norfloxacin transport on the lipid composition of the membrane, and in particular investigated bilayers containing different types of lipid molecules commonly found in the cytoplasmic membrane of *Escherichia coli*^17^. Our technique also enables direct measurements of drug uptake in proteoliposomes containing the *E. coli* outer membrane protein OmpF in the membrane; we thus quantified the role of these porins in norfloxacin transport^18^.

It is important to note here that bulk permeability measurement techniques such as the parallel artificial membrane permeability assay (PAMPA) need to account for the effect of the unstirred water layer (UWL) on drug permeation^19^,^20^. This aqueous layer may range up to hundreds of microns in size, and this additional boundary layer may replace the lipid membrane as the rate limiting step for drug permeation^7^,^19^. In this layer, there exists a concentration gradient of the drug. In our vesicle based assay, however, we can directly observe the concentration of the drug surrounding the vesicles via the autofluorescence of the drug molecules. In our previously published work, we imaged the cross section of a vesicle suspended in a bath of norfloxacin in a confocal microscope; we found no evidence for a large (micron scale) UWL surrounding the vesicles^4^. Similar single vesicle fluorescence experiments were also previously performed to study the transport (or lack thereof) of indole and indole acetic acid across lipid vesicle membranes. In these experiments as well there was no evidence to suggest a large UWL surrounding the vesicles^21^.

This evidence does not rule out the possibility of having a nanoscale UWL around the vesicles. However, the effect of the UWL on drug permeation is expected to be a function of velocity^22^, and we have previously shown that the permeability coefficients measured in our assay are independent of vesicle velocity^4^. Finally, we have used our assay to study the role of lipid composition on drug transport; we observed that simply changing the lipid molecules in the vesicle membranes from DPhPC to DOPC changed drug permeability by over an order of magnitude^17^. Thus the lipid membrane appears to be the rate limiting step to drug permeation in our assay. Even if a nanoscale UWL does exist around the vesicles, its effect on drug permeation appears to be minimal, and may be neglected here.

In this paper, we performed measurements using phosphatidylcholine (PC) lipids, which stably and reproducibly form vesicles via electroformation. Our goal was simply to extend our technique to the study of different fluoroquinolones; we have already studied the effect of lipid composition on drug transport^17^. We measure the permeability of enrofloxacin, pefloxacin and fleroxacin at three different physiologically relevant pH values, corresponding to different charge states of the drug molecules^3^. A comparison of these molecules with norfloxacin reveals differences in their lipid permeation capabilities that span over two orders of magnitude.

We approach the problem of drug uptake in bacteria from a reductionist perspective, and show that the study of even simple PC lipids can help us understand the significant permeation differences between these fluoroquinolones. Phosphatidylcholine is also an important constituent of the intestinal mucus layer^23^. Thus our measurements might be applicable to the understanding of drug absorption in the intestine as well.

The technique can be extended to all fluorescent drugs/molecules, thus providing quantitative information about the transport of these drugs across lipid bilayers with well controlled compositions. We also discuss improvements in the experimental procedure and present a more streamlined analysis script which should make the measurement technique more accessible to other researchers in the field.

## Materials and Methods

**Figure 1:**
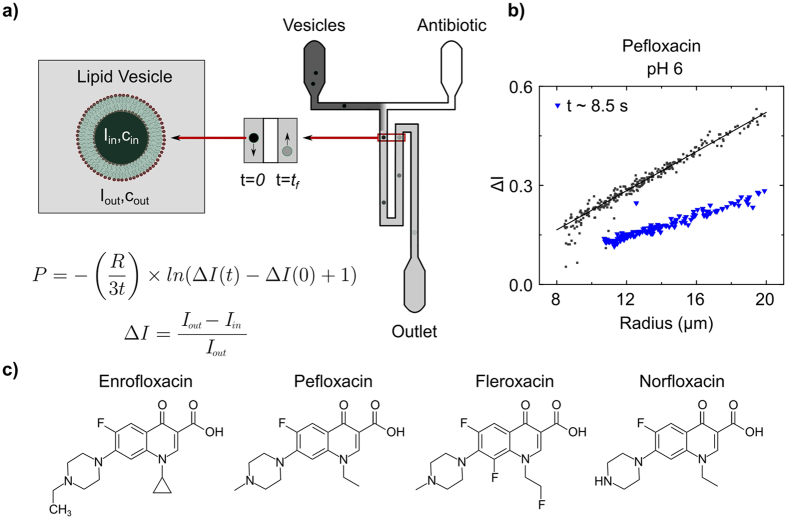
Schematic of the optofluidic assay. (**a**) Vesicles and antibiotic molecules are mixed using a T junction microfluidic chip. Under UV illumination, the antibiotic molecules are autofluorescent. The channel width is 40 μm. Vesicles can be detected at two time points in the same field of view. As a vesicle traverses the channel, the drug permeates into the vesicle and hence the drug autofluorescence intensity within the vesicle increases. This increase in drug autofluorescence inside the vesicle (I_in_) is used to determine the permeability coefficient of the drug across the vesicle lipid membrane on the basis of a simple diffusion model^4^. (**b**) Typical experimental data, depicting the permeation of pefloxacin into DPhPC lipid vesicles at pH 6. Each point in the scatter plot represents an individual vesicle. The dark grey squares refer to vesicles detected at the initial (t = 0) time point whereas the blue triangles refer to vesicles detected at the later detection point (on average 8.5 s further downstream for this particular measurement). The downward shift in *Δ*I with time is a result of the increase in autofluorescence within the vesicles (I_in_) and is thus a direct measurement of pefloxacin permeation into the vesicles. Similar scatter plots for the other drug/pH conditions measured in this paper are presented in the Supplementary Information. (**c**) Chemical structures of the fluoroquinolone antibiotics investigated in this paper arranged in order of decreasing octanol partition coefficients (Table 1).

The T junction microfluidic chips used in the experiments (shown schematically in Fig. 1a) were constructed using standard photo- and soft lithography techniques^4^,^18^,^24^. The chips are made of Sylgard 184 polydimethylsiloxane (PDMS, Dow Corning) using an elastomer : curing agent ratio of 9:1. The chips are designed such that two time points may be observed in the same field of view (60× magnification). We have constructed multiple designs with different lengths of the mixing channel, which enable us to observe the vesicles over different timescales. For fast permeation experiments (on the order of a few seconds), we used a chip design where the channel length from the T junction to the outlet reservoir was 28 mm^18^. For measurements where the permeation was found to be slower (minutes), we used a chip with a channel length of approximately 380 mm from the T junction to the outlet reservoir^4^. In our experiments, vesicles typically flow through the channel at speeds of around 1 mm/s. AutoCAD drawings of the different designs are available on request.

Unless otherwise stated, all chemicals were obtained from Sigma Aldrich. 5 mM phosphate buffers were used for experiments at pH 6, pH 7 and 5 mM acetic acid as the buffer at pH 5. 1,2-diphytanoyl-*sn*-*glycero*-3-phosphocholine (DPhPC, Avanti Polar Lipids) lipid vesicles were prepared in the respective buffers in the presence of 200 mM sucrose using a standard electroformation protocol as previously described^4^.

In the experiments, 2 mM solutions of the antibiotics were introduced at one of the inlets of the microfluidic chip, with the other inlet containing the vesicles. The antibiotic solutions were prepared in the relevant buffer/sucrose solutions and the pH adjusted to desired values using HCl/KOH. Suction was applied at the outlet reservoir to pull vesicles and antibiotic molecules into the mixing channel. As shown in the schematic (Fig. 1a), once past the T junction, the antibiotic molecules diffuse across the width of the channel. The vesicles thus pass through a bath of drug molecules, and as the drug diffuses across the vesicle membrane, the drug autofluorescence intensity within the vesicle increases. By studying this increase in autofluorescence within the vesicles over time, the permeability coefficient (*P*) of the drug across the lipid membrane can be extracted using the following equation^4^:


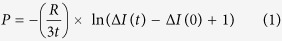


where *R* is the vesicle radius, *t* is the time taken for the vesicle to move from the initial (*t* = 0) to the final (*t*) detection point, and Δ*I* is the normalised drug autofluorescence intensity difference between the interior (*I*_*in*_) and the exterior (*I*_*out*_) of the vesicle:


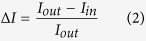


As the drug permeates into the vesicle, *I*_*in*_ increases and hence Δ*I* decreases. Thus, the decrease in Δ*I* for the vesicles over time is a direct measure of drug permeation into the vesicles. The permeability equation above is designed to account for the fact that the experiment uses brightfield rather than confocal fluorescence microscopy. The consequences of such an experimental setup for the measurement of drug fluorescence within a vesicle are explained in detail in the ESI of ref. 4. In addition, we have provided comprehensive explanations of the assumptions inherent in the permeability calculation in the supplementary information attached with this paper.

The optofluidic assay is performed as follows:

- The PDMS chip is bonded to a glass coverslip (type I thickness, Assisstent) by a standard plasma bonding procedure using an air plasma (10 W, 25 sccm, 10 s exposure, Diener Electronic GmbH & Co. KG). The bonded chip is then left in an oven at 60 °C for 5–10 mins to enhance the adhesion. Following this, chips are immediately filled with buffer solution to wet the channels.
- A pipette tip containing 40 μl of the antibiotic solution is inserted into one inlet column, and similarly a pipette tip containing 40 μl of the vesicle solution is inserted into the other inlet column.
- Suction is applied at the outlet using a neMESYS syringe pump system with a 250 μl Duran Borosilicate glass syringe connected to the chip using Upchurch 1520 G tubing.
- The chip, with its connections, is placed on the stage of a UV epifluorescence microscope containing a 60× objective. We performed most of the experiments described in this paper on a custom-built microscope^4^ with an excitation wavelength of 340 or 350 nm, selected using a monochromator (Oceanoptics Monoscan 2000); an EQ99FC (Energetiq) broadband white light source was used. However, we also performed experiments using a commercial Olympus IX73 microscope with a DAPI filter set (Chroma) and an arc lamp (Prior Lumen 200) as the light source. There was no significant difference observed in the results with the different excitation wavelengths, as expected; any standard DAPI fluorescence setup is therefore suitable for our technique.
- Initially, a high flow rate is applied (30–50 μl/hr). Once the drug fluorescence intensity reaches its peak value in the channel, the flow rate is lowered to 3 μl/hr.
- Once the flows stabilise, images are recorded at 100 fps. We used an Evolve 512 Delta EMCCD (Photometrics) or an optiMOS scientific CMOS camera (QImaging) in our experiments. Binning reduces the size of the data being acquired, averages over fluctuating pixels and optimises the frame rate. Images were recorded using μManager 1.4^25^.
- The permeability coefficient of the drug is inferred from the increase in drug autofluorescence intensity within the vesicles over time^4^.
- Image analysis is performed using MATLAB scripts as described previously^4^,^18^. Briefly, vesicles are tracked for multiple frames, from which individual vesicle velocities can be calculated. Combined with a knowledge of the chip geometry, one can calculate the time (*t*) taken by each vesicle to move from the initial to the final detection position. The radius of a vesicle is calculated as the average of the semi-major and semi-minor axes of the detected shape. *I*_*in*_ is measured around the centre of the vesicle, and *I*_*out*_ is measured at the same location in the absence of the vesicle (basic versions of the MATLAB scripts used are freely available as part of the ESI of ref. 4). We have also developed a new MATLAB user interface for the final data analysis; this is described in the Supplementary Information document and the script is attached. A detailed description of the filters applied is also provided in the Supplementary Information.

## Results and Discussion

**Figure 2:**
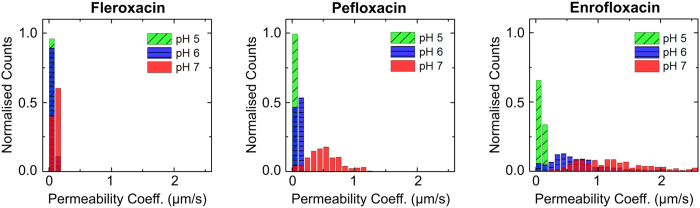
Histograms representing the measured permeability coefficients of fleroxacin, pefloxacin and enrofloxacin across DPhPC lipid vesicle membranes as a function of pH. The counts provided here are normalised to the total number of vesicles detected in each case, to enable direct comparison of the permeability coefficients across the different conditions. For all three fluoroquinolones, the permeability increases as we increase the pH from acidic (pH 5) to neutral values. The mean permeability coefficients are reported in Table 1. From the histograms, we observe that fleroxacin has the lowest permeability of the three drugs, and enrofloxacin the highest. Separate histograms and the experimental scatter plots for each condition are provided in the Supplementary Information.

Experiments were performed at pH 5, 6 and 7, corresponding to different charge states of the fluoroquinolones being investigated (Fig. 2). For all three drugs, significant lipid permeation was observed within the timescales investigated for all three pH values. At pH 6 and pH 7, enrofloxacin transport was so rapid that the second detection point had to be chosen less than a second away from the first detection point in the flow system; at later times the contrast between the vesicles and the background was too low for them to be detected using this technique, since our analysis detects vesicles based on their contrast with the background fluorescence. A similar level of transport was observed with pefloxacin at pH 7, as seen in the relevant scatter plots in Figures S4 and S6.

**Figure 3:**
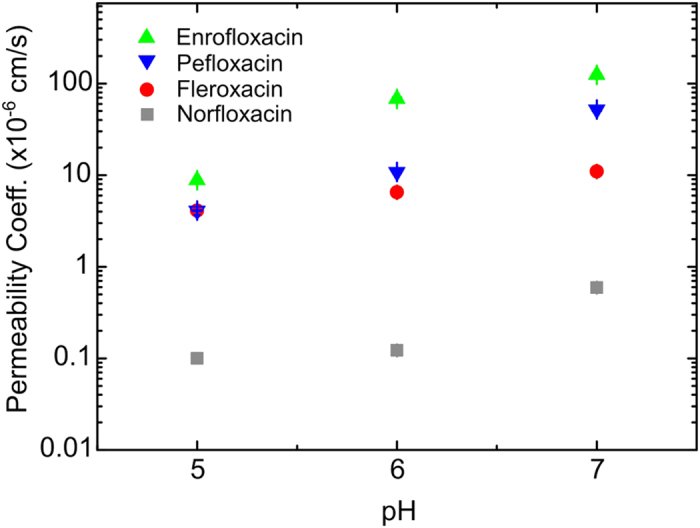
Comparison of the permeability coefficients (P) for the four fluoroquinolones studied as a function of pH. The error bars (s.e.) are smaller than the symbol size. The permeability coefficient values for norfloxacin at pH 5 and 7 are taken from Ref. 4. For all the molecules, the permeability increases as we increase the pH from 5 to 7. We find large differences in the absolute values of the permeability coefficients between the drugs; the permeability of enrofloxacin is approximately two orders of magnitude greater than that of norfloxacin, across the pH range studied. Norfloxacin has the lowest permeability of the drugs studied, while enrofloxacin has the highest.

For all three drugs, we observed an increase in permeability coefficients as we increased the pH from 5 to 7. The permeability coefficients are provided in Table 1, and depicted visually in Fig. 3. Histograms of the permeability coefficients (from all the experiments performed) are provided in Fig. 2 and in further detail in Supplementary Figure S7. In our earlier work we had measured the permeability of norfloxacin at pH 5 and 7; for completeness, we measured norfloxacin permeability at pH 6 as well (Supplementary Information Figure S3). At pH 5, the molecules are predominantly positively charged, whereas the proportion of zwitterionic/uncharged molecules is higher at pH 6 and 7. This explains the pH dependence of the permeability coefficients, since charged moieties are less lipophilic as they do not easily dissolve in the hydrocarbon core of the lipid bilayer.

The four molecules studied are all fluoroquinolone derivatives, but differ with respect to the structure of their chemical substituents. It is these subtle differences in the substituents that underpin the wide range of permeability coefficients (Fig. 3). All four derivatives contain a piperazin-1-yl group at the C-7 position (see Supplementary Figure S2 for the general chemical structure). However, only in the norfloxacin molecule is the amine at the N-4 position of the piperazin-1-yl group a secondary amine. In the other three molecules, these amines are tertiary and therefore their hydrophobicity is enhanced compared to norfloxacin^26^. This explains the lower lipid solubility of norfloxacin; it therefore has the lowest permeability value of the series. At the other end of the spectrum, enrofloxacin exhibits the largest permeability value. This compound possesses a 4-ethyl-piperazin-1-yl group at the C-7 position that confers more lipophilicity than the 4-methyl-piperazin-1-yl present in fleroxacin and pefloxacin, as evidenced by the corresponding octanol partition coefficients of the different molecules (Table 1). This derivative also contains a cyclopropyl group at N-1 whereas the other molecules contain an ethyl group at the same position. However, the difference in lipophilicity conferred by these two substituents at N-1 is reported to be less significant compared to the effect of increasing the hydrophobic character of the C-7 substituent^26^. It is therefore likely that the 4-ethyl-piperazin-1-yl C-7 substituent of enrofloxacin confers it with the highest permeability value of the series.

Fleroxacin and pefloxacin were found to have similar permeability at pH 5, with the permeability of pefloxacin being higher at pH 6 and pH 7. Fleroxacin has two extra fluorine atoms compared to pefloxacin, one attached to the aromatic ring at C-8 and the other in the ethyl group at N-1. The fluorine atom on the aromatic ring is expected to increase the lipophilicity^27^, but the fluorine atom in the aliphatic group has been shown to produce the opposite effect^28^. The latter may be responsible for the reduced lipophilicity and hence the decreased permeability value of fleroxacin. This also explains the higher octanol partition coefficient of pefloxacin compared to fleroxacin (Table 1).

## Conclusions

In this paper we extended our optofluidic measurement technique to investigate differences in the permeability of four fluoroquinolones over a range of pH values. The results obtained are validated by considering the chemical structures of the molecules; the permeability coefficients increase with an increase in the lipophilicity of the molecules, as expected. This technique can complement traditional drug transport assays such as the parallel artificial membrane permeability assay (PAMPA)^29^ as we directly measure the permeability coefficient of the drug across the lipid membrane of interest. Furthermore, unlike earlier techniques, we have shown that this assay can be used to study drug uptake through transporters^18^; the technique can thus be used to investigate drug transport across more realistic model membranes, containing different membrane proteins commonly found in bacterial cell membranes. To extend the technique to molecules that are not autofluorescent, reporter complexes that fluorescence upon binding to a drug molecule could be used; such fluorescent drug-reporter complexes have been studied previously in vesicles^14^. Finally, a number of new microfluidic vesicle preparation techniques are under development^30^,^31^. Such technologies could be combined with our assay to make the entire process, from vesicle production to drug testing, functional on a single microfluidic device. We believe that such developments will be of great benefit to all those interested in studying drug-membrane interactions in both the academic and pharmaceutical communities.

## Additional Information

**How to cite this article**: Cama, J. *et al.* Direct Optofluidic Measurement of the Lipid Permeability of Fluoroquinolones. *Sci. Rep.* **6**, 32824; doi: 10.1038/srep32824 (2016).

## Supplementary Material

Supplementary Information

Supplementary Movie

Supplementary Information

## Figures and Tables

**Table 1:** Permeability Coefficients (×10^−6^ cm/s, mean ± s.e).

Antibiotic	pH 5	pH 6	pH 7	Literature Values of Apparent Octanol Partition Coefficients (log K_app_, mean ± s.d)
Enrofloxacin	8.8 ± 0.1	68 ± 3	124 ± 4	3.48 ± 0.04 (pH 7)^32^
Pefloxacin	4.11 ± 0.05	10.8 ± 0.2	52 ± 2	0.23 ± 0.05 (pH 7)^33^
Fleroxacin	4.1 ± 0.2	6.5 ± 0.1	11.0 ± 0.2	−0.72 ± 0.18 (pH 7.4)^34^
Norfloxacin	0.1	0.122 ± 0.004	0.59 ± 0.02	−1.00 ± 0.03 (pH 7)^33^
