# Supplementary material for: Direct Optofluidic Measurement of the Lipid Permeability of Fluoroquinolones
Source: Sci Rep. 2016 Sep 8;6:32824. doi: 10.1038/srep32824 (PMC5015079; doi:10.1038/srep32824)
Supplement: Supplementary Information [file srep32824-s1.pdf]

Direct Optofluidic Measurement of the Lipid Permeability of Fluoroquinolones

Jehangir Cama, Michael Schaich, Kareem Al Nahas, Silvia Hernández-Ainsa, Stefano Pagliara, Ulrich F. Keyser

SUPPLEMENTARY INFORMATION

1. Updated Data Analysis Technique

The measurement of the permeability coefficient is based on a simple model that considers the diffusion of antibiotic molecules across the lipid bilayer of a spherical vesicle when it is suspended in a bath of the drug. Diffusion is driven by the drug concentration gradient between the exterior (c_{out}) and the interior (c_{in}) of the vesicle. Since we track the concentration of the drug molecules using their autofluorescence, we can relate the drug concentrations to the autofluorescence intensities outside (I_{out}) and inside (I_{in}) the vesicle. After solving the diffusion model, and accounting for the experimental setup being a bright-field rather than a confocal microscope, we arrived at an equation for the permeability coefficient (P) that depends exclusively on experimentally measured parameters (full details of the model are available in the freely accessible Supplementary Information document of Ref. 1):

$$P = -\left(\frac{R}{3t}\right) \times \ln(\Delta I(t) - \Delta I(0) + 1) \qquad (1)$$

where, as mentioned in the main manuscript, R refers to the vesicle radius, t is the time taken between initial and final detection points, and ΔI is a normalised autofluorescence intensity difference between the exterior and interior of the vesicle:

$$\Delta I = \frac{I_{out} - I_{in}}{I_{out}} \qquad (2)$$

$\Delta I(0)$ refers to the value measured at the initial detection point, and $\Delta I(t)$ is the value measured at the later detection point. In our earlier work (ref. 1) we confirmed the exponential nature of the diffusion process, and proved that the model is an accurate representation of drug permeation across the vesicle membrane.

The initial image analysis scripts, used to analyse the raw images acquired, are freely available in the ESI of ref. 1; these scripts formed the basis of the data analysis for this paper, with a few modifications for improved vesicle detection efficiency. The final analysis is now performed using our new MATLAB graphical user interface (GUI), whose script is attached with this paper. The GUI allows for the quick visualisation of the scatter plots, and is an easy to use platform where the filters can be adjusted as per requirements as described below.

The time dependence in the equation for P is due to the fact that we are studying the accumulation of drug molecules in a vesicle, which naturally has to occur over a finite amount of time. In the model, we also assumed (based on preliminary, single vesicle experiments[1]) that once past the lipid bilayer, the drug equilibrates almost instantly within the vesicle. In reality, this equilibration of the drug molecules within the vesicle will take a finite amount of time, which we estimate to be on the order of 70 ms for a vesicle of radius 10 μm. Thus the model is not applicable at time scales shorter than around 100 ms.

We apply filters on the radius and velocity as before[1,2] such that only vesicles with velocities between 0.4 – 1.5 mm/s are selected for analysis. The variability in vesicle speed is a direct

consequence of the parabolic velocity profile of the fluid, which is a result of the pressure driven fluid flow exerted by the syringe pump in the microchannel (for a theoretical discussion of the relevant formulae, the reader is referred to *Theoretical Microfluidics* by Henrik Bruus, Oxford University Press, 2009 Reprint, Section 3.4). Smaller vesicles can flow through the channel in different positions, whereas larger vesicles are constrained to flow through the centre of the channel. Thus the smaller vesicles are bound to show variability in speed, depending on their position in the channel. From experience, we found that for velocities lower than 0.4 mm/s, the vesicles were found to occasionally roll along the channel walls – the velocity therefore may not be uniform, and we discard such events. Detections at speeds faster than 1.5 mm/s were generally false positives, and the images were also subject to blurring which is why these were neglected. Vesicles with radii between approximately 8 – 20 μm were chosen for analysis. Vesicles with radii greater than 20 μm were subject to shear along the channel walls (channel width = 40 μm), while vesicles with radii less than about 8 μm were difficult to distinguish from lipid aggregates due to their small size. Furthermore, at small radii, the vesicles have more vertical space in the channel to explore in the flow (the channel heights were 43 and 50 μm for the short and long chip designs respectively) and hence were more likely to be out of focus than larger vesicles. Due to this, the ΔI values for vesicles with smaller radii are occasionally noisy. To prevent this from biasing individual experiment results, we fit the linear relation between $\Delta I(0)$ and R using points with $R > 12$ μm and extend the fit over the R axis. Since this fit is used to calculate $\Delta I(t) - \Delta I(0)$ in equation (1), it is important to ensure that there is no bias due to noise in the $\Delta I(0)$ values at low R. Our new MATLAB GUI allows the user to select the cutoff value of R for the fit after observing the spread in the distribution ("**Radius (um) Threshold for DI(0) fit**" in the GUI). All the other filter parameters can also be adjusted using the GUI (note that the upper and lower radius filters are provided in pixels). To operate the GUI, we load the csv files from our earlier 'postprocess' code[1]; ROI1 and ROI2 refer to vesicles in the two detection regions of interest (i.e, the two different time points, see Figure S8) in the images. The distance between detection points (ROIs) can also be selected based on a knowledge of the chip geometry. Finally, an option for fitting the histograms with a normal or log-normal distribution is also provided, although we have not used this in our data analysis; we simply report the mean value of the permeability coefficients measured over all the vesicles.

Another consideration to take into account is that we detect the vesicles based on their contrast with the drug autofluorescence background in the channel. As the drug concentration inside a vesicle increases, the flux of molecules into the vesicle decreases and the difference in the autofluorescence intensities inside the vesicle when viewed at later time points becomes lower and lower. Beyond a certain timeframe, these differences will not be detectable since the signal to noise ratio (SNR) will be highly diminished as I_{in} approaches I_{out}. Therefore, the timeframe within which measurements should be performed must be optimised. To this end, we first apply a filter on the ΔI values; we delete all points with $\Delta I < 0.05$, since for these points the SNR is found to be too low to distinguish true vesicle events from lipid aggregates or other false positives (in the GUI, this cutoff is chosen using the "**DI(t) cutoff value**" slider).

Finally, after applying the velocity, radius and ΔI filters, we calculate the permeability coefficient as per equation (1). We then used the permeability coefficient determined in the previous step, along with the $\Delta I(t)$ cutoff at 0.05, to determine the upper time limit of the measurement (t_f):

$$t_f = -\left(\frac{R}{3P}\right) \times \ln(0.05 - \Delta I(0) + 1) \quad (3)$$

We used this to delete the vesicles for which $t > t_f$. This was done iteratively till all the vesicles in a measurement fell within the t_f boundary. An example of this is provided in Figure S1. The red circles represent the $\Delta I(t)$ values for vesicles that remained after the initial radius, velocity

and ΔI filters were applied. The blue circles represent the vesicles that remain after the t_f filter is applied. It is these vesicles that are used to calculate the final, reported permeability value. This feature is built into the GUI as well (although after analysing and exporting the data, both datasets are provided, one in which the t_f filter is applied and the other where it isn't). We believe that this new analysis protocol makes the technique more robust to experimental noise and also provides a quantitative explanation of the optimum timescales for the measurement. This optimum timeframe is ultimately determined by the vesicle detection sensitivity of the optical setup, and depends on how well vesicles can be detected via their contrast against the drug autofluorescence background.

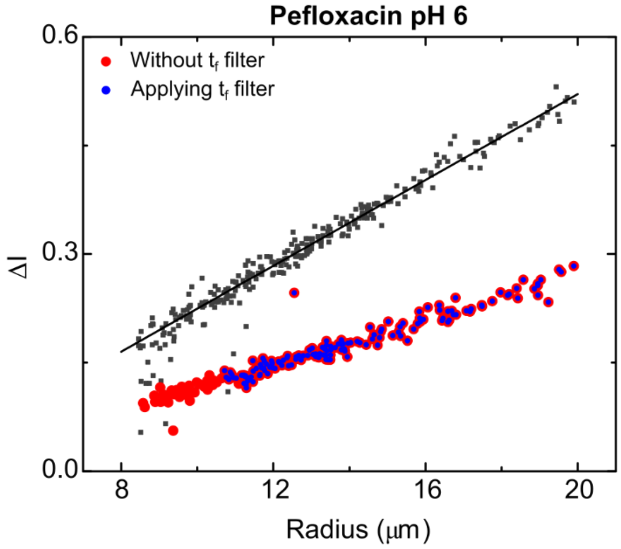

Figure S1. An example of the new filtering technique that deletes vesicles which fall outside the optimal detection time for a measurement. After obtaining a first estimate of the permeability coefficient, this value is used to determine the upper time limit in which the permeability measurement should be performed, as a function of radius, based on our experimental detection limits. Vesicles for which the time t between initial and final detection was greater than t_f are discarded and the process repeated iteratively till all the remaining vesicles satisfy $t<t_f$. In the scatter plot shown, the red circles represent the vesicles before applying the filter, and the blue circles represent the final selection that satisfy the criterion. Only those vesicles that satisfy the criterion are used for the final determination of P.

2. General chemical structure of the fluoroquinolones studied

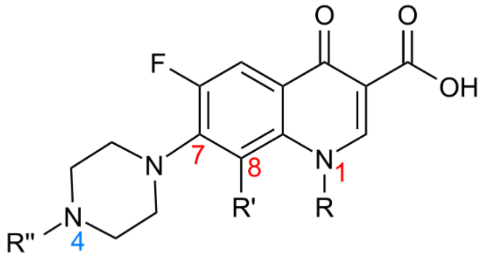

Figure S2. General chemical structure of the fluoroquinolones studied in this paper. The numbers assigned are consistent with the explanation provided in the main text. Note that red numbers are used for labelling the atoms contained in the main fluoroquinolone structure (N-1, C-7 and C-8), whereas blue is used to identify the nitrogen (N-4) of the piperazin-1-yl substituent of the fluoroquinolones at C-7.

3. Experiment Datasets

The data for the norfloxacin permeability measurement at pH 6 is provided in Figure S3 below. Since the permeation process was much slower than for the other drugs, a longer chip design[1] had to be used. Vesicles were detected about 5 minutes (on average) after the initial detection location, at which point significant drug permeation could be observed as seen in the decreased ΔI values in Figure S3.

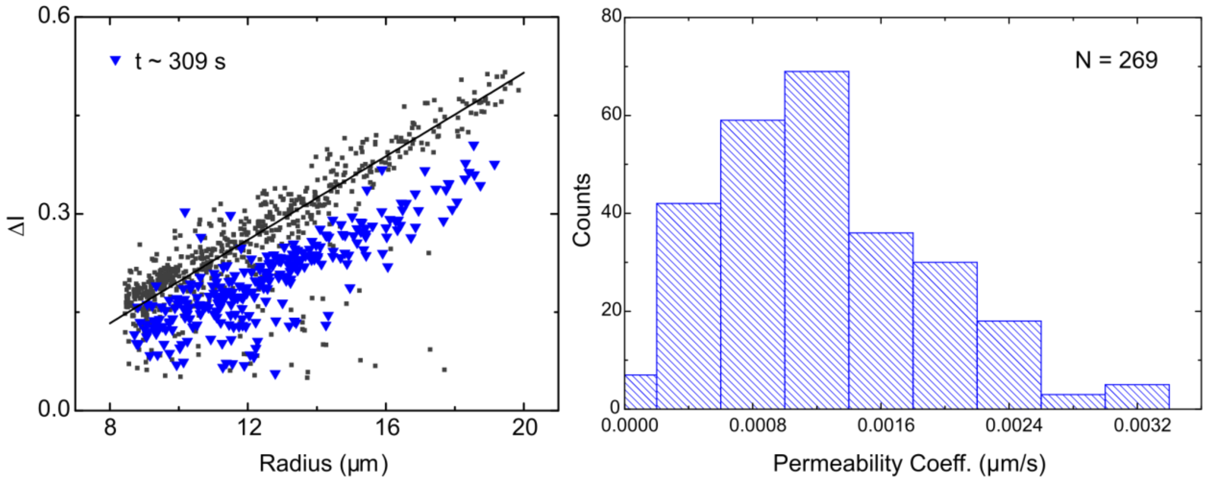

Figure S3. Norfloxacin permeability measurements at pH 6. The experiment was performed over a timescale of approximately 5 minutes, since norfloxacin permeation across DPhPC membranes was found to be much slower than the other fluoroquinolones studied in this paper. The histogram shows the spread in the permeability values measured, and shows the typical spread about the mean value. The permeability coefficient was determined to be $1.22 \pm 0.04 \times 10^{-7}$ cm/s (mean ± s.e, N = 269).

The scatter plots of the experimental repeats for pefloxacin, fleroxacin and enrofloxacin are provided in Figure panels S4, S5 and S6. The detailed histograms summarising data from all the experiments are provided in Figure panel S7.

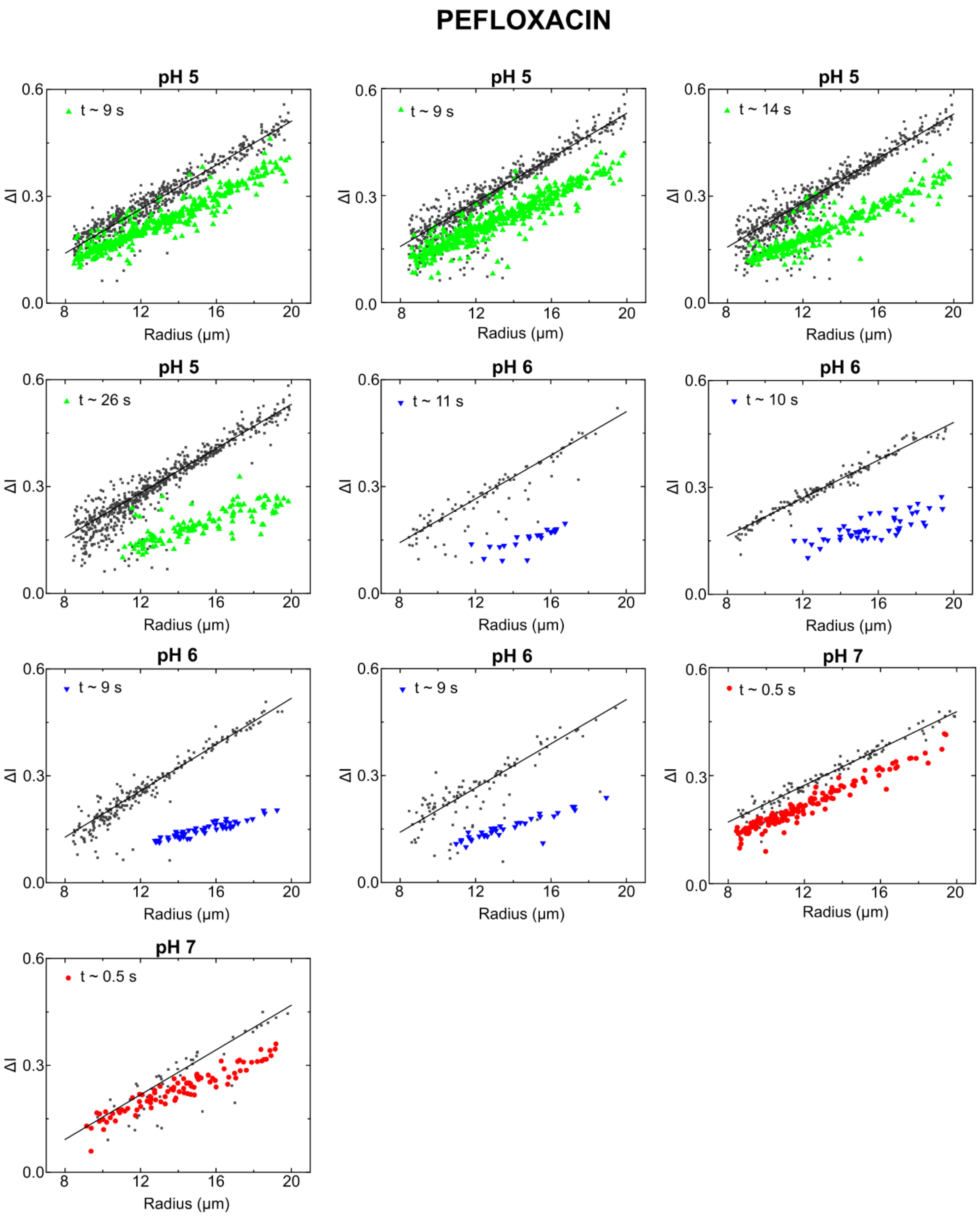

Figure S4. Scatter plots of ΔI vs R of experimental repeats for pefloxacin under different pH conditions. All the plots present data after all the filters have been applied. In each plot, significant drug transport is observed in a majority of the vesicles detected.

FLEROXACIN

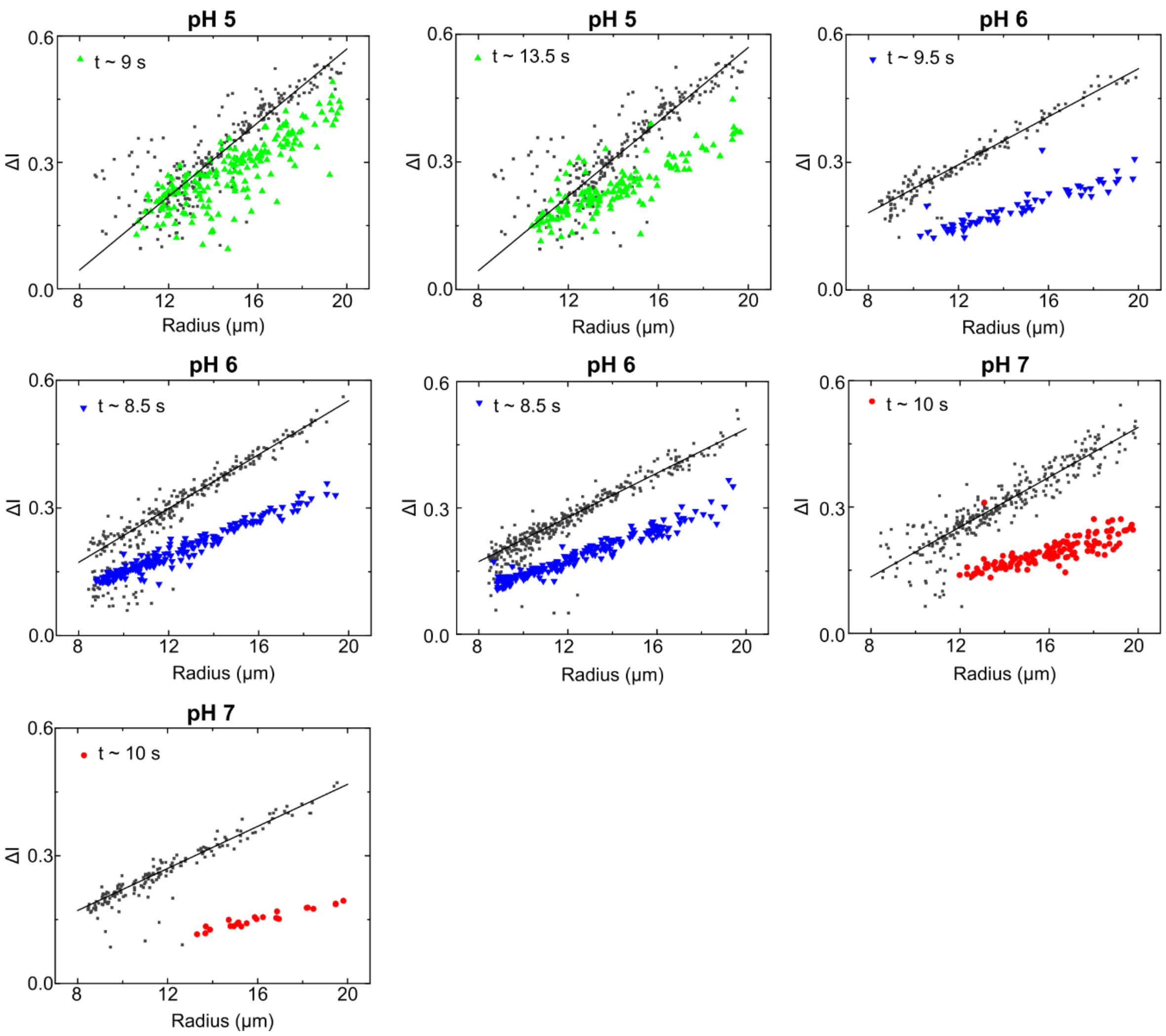

Figure S5. Scatter plots of ΔI vs R of experimental repeats for fleroxacin under different pH conditions. All the plots present data after all the filters have been applied. In each plot, significant drug transport is observed in a majority of the vesicles detected.

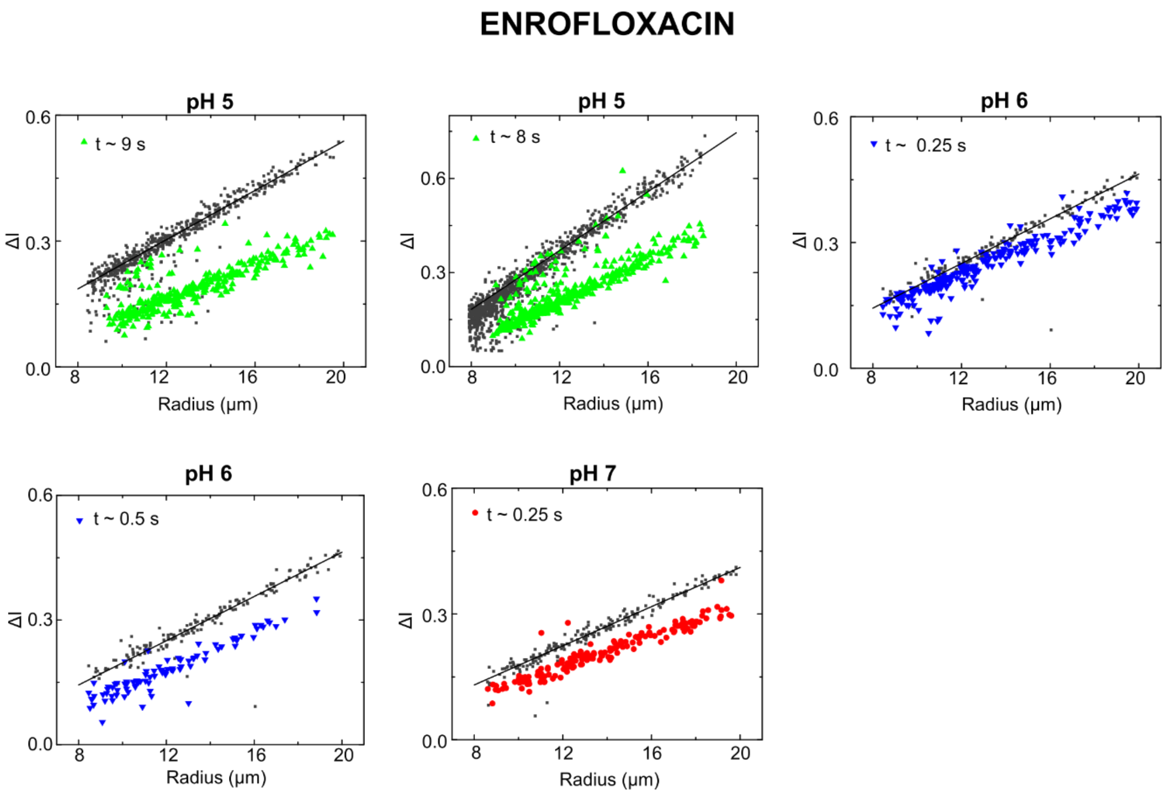

Figure S6. Scatter plots of ΔI vs R of experimental repeats for enrofloxacin under different pH conditions. All the plots present data after all the filters have been applied. In each plot, significant drug transport is observed in a majority of the vesicles detected.

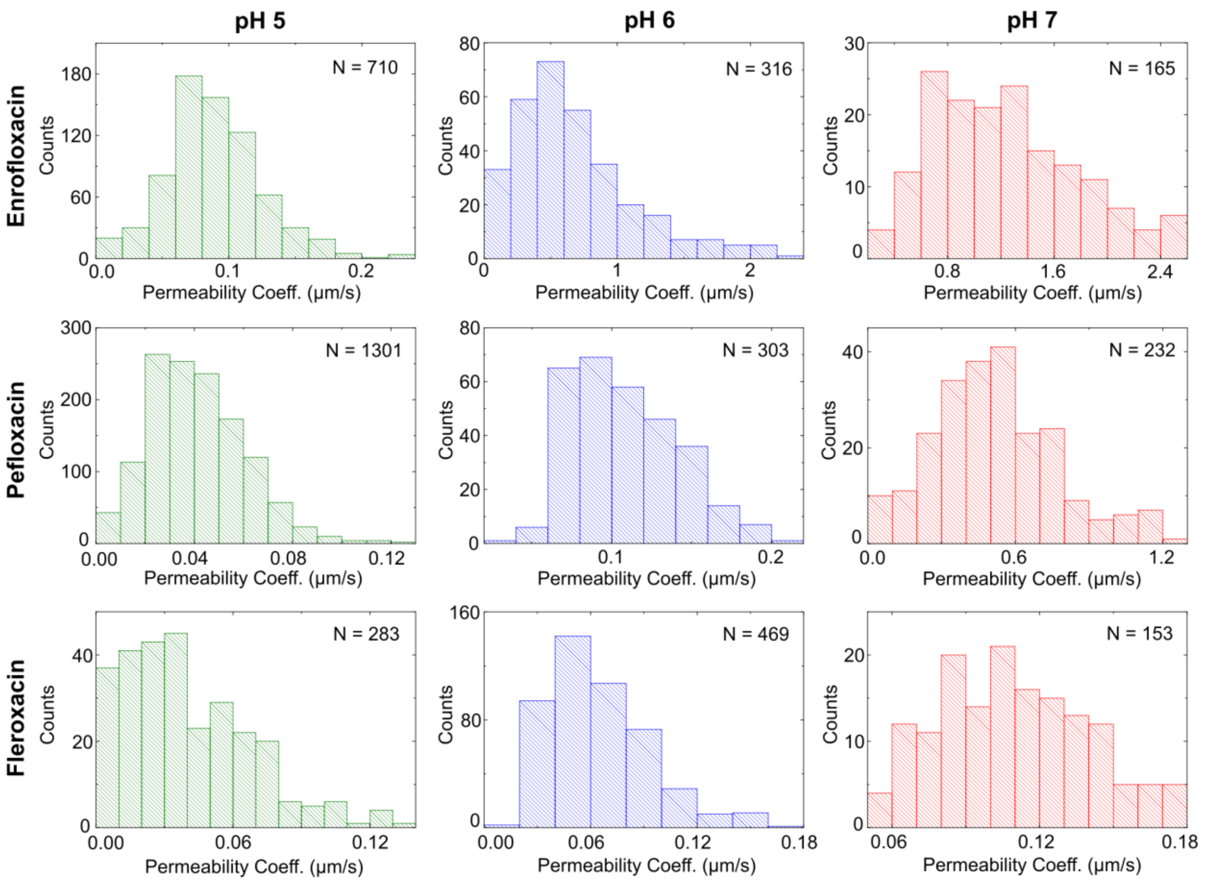

Figure S7. Permeability coefficient histograms for enrofloxacin, pefloxacin and fleroxacin at pH 5,6 and 7. The histograms contain permeability measurements from all the different experimental repeats. The number of vesicles detected generally increases as the pH is decreased, since electroformation yields were higher at the lower pH values. The total numbers of vesicles (N) analysed across all experimental repeats are provided inset within each histogram.

4. Typical Vesicle Detection Image

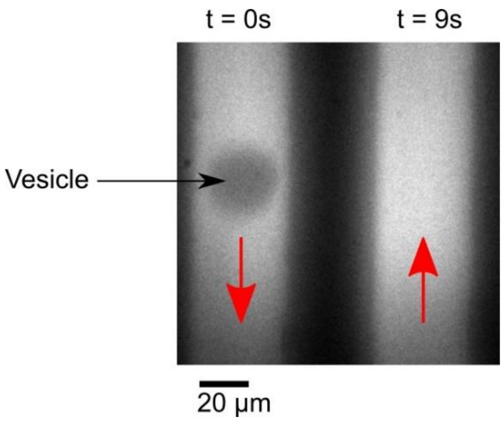

Figure S8. Typical vesicle detection field of view in an experiment. The drug (pefloxacin, pH 6) is fluorescent under UV illumination. One observes that the microchannel is filled with the drug, and the fluorescence pattern shows that the drug molecules have diffused across the width of the channel by the initial detection location (t = 0s). An AVI file of typical vesicle detections is attached, where one can observe the increase in drug fluorescence intensity within vesicles as they traverse the microchannel. The orientation of the AVI file is the same as this figure. The red arrows depict the direction of flow.

5. Description of Video File

Video microscopy of DPhPC vesicles as they traverse the microfluidic channel containing pefloxacin at pH 6. The time taken for the vesicles to travel between the two detection locations is approximately 9 s. The vesicles show a clear increase in internal fluorescence intensity at the later detection point, thus showing the accumulation of pefloxacin within the timescales measured. For clear visualisation, the video is slowed down 5x compared to the detection frame rate.

References

1. Cama, J., Chimerel, C., Pagliara, S., Javer, A. & Keyser, U. F. A label-free microfluidic assay to quantitatively study antibiotic diffusion through lipid membranes. *Lab Chip* **14,** 2303–2308 (2014).

2. Cama, J. *et al.* Quantification of Fluoroquinolone Uptake through the Outer Membrane Channel OmpF of Escherichia coli. *J. Am. Chem. Soc.* **137,** 13836–13843 (2015).

Open Data Statement

All data accompanying this publication are directly available within the main manuscript or in the supporting information of this publication.
